# Mobile for Mothers mHealth Intervention to Augment Maternal Health Awareness and Behavior of Pregnant Women in Tribal Societies: Randomized Quasi-Controlled Study

**DOI:** 10.2196/38368

**Published:** 2022-09-21

**Authors:** Avishek Choudhury, Murari Choudhury

**Affiliations:** 1 Industrial and Management Systems Engineering Benjamin M Statler College of Engineering and Mineral Resources West Virginia University Morgantown, WV United States; 2 Network for Enterprise Enhancement and Development Support Deoghar India

**Keywords:** maternal health, mHealth, digital divide, disparity, socioeconomic, India, health, awareness, mobile, intervention, adherence, health behaviors, tribal, community, education

## Abstract

**Background:**

Despite several initiatives taken by government bodies, disparities in maternal health have been noticeable across India’s socioeconomic gradient due to poor health awareness.

**Objective:**

The aim of this study was to implement an easy-to-use mobile health (mHealth) app—Mobile for Mothers (MfM)—as a supporting tool to improve (1) maternal health awareness and (2) maternal health–related behavioral changes among tribal and rural communities in India.

**Methods:**

Pregnant women, aged 18 to 45 years, were selected from two rural villages of Jharkhand, India: (1) the intervention group received government-mandated maternal care through an mHealth app and (2) the control group received the same government-mandated care via traditional means (ie, verbally). A total of 800 accredited social health activists (ASHAs) were involved, of which 400 were allocated to the intervention group. ASHAs used the MfM app to engage with pregnant women during each home visit in the intervention group. The mHealth intervention commenced soon after the baseline survey was completed in February 2014. The end-line data were collected between November 2015 and January 2016. We calculated descriptive statistics related to demographics and the percentage changes for each variable between baseline and end line per group. The baseline preintervention groups were compared to the end-line postintervention groups using Pearson chi-square analyses. Mantel-Haenszel tests for conditional independence were conducted to determine if the pre- to postintervention differences in the intervention group were significantly different from those in the control group.

**Results:**

Awareness regarding the five cleans (5Cs) in the intervention group increased (*P*<.001) from 143 (baseline) to 555 (end line) out of 740 participants. Awareness about tetanus vaccine injections and the fact that pregnant women should receive two shots of tetanus vaccine in the intervention group significantly increased (*P*<.001) from 73 out of 740 participants (baseline) to 372 out of 555 participants (end line). In the intervention group, awareness regarding the fact that problems like painful or burning urination and itchy genitals during pregnancy are indicative of a reproductive tract infection increased (*P*<.001) from 15 (baseline) to 608 (end line) out of 740 participants. Similarly, knowledge about HIV testing increased (*P*<.001) from 39 (baseline) to 572 (end line) out of 740 participants. We also noted that the number of pregnant women in the intervention group who consumed the prescribed dosage of iron tablets increased (*P*<.001) from 193 (baseline) out of 288 participants to 612 (end line) out of 663 participants.

**Conclusions:**

mHealth interventions can augment awareness of, and persistence in, recommended maternal health behaviors among tribal communities in Jharkhand, India. In addition, mHealth could act as an educational tool to help tribal societies break away from their traditional beliefs about maternal health and take up modern health care recommendations.

**Trial Registration:**

OSF Registries 9U8D5; https://doi.org/10.17605/OSF.IO/9U8D5

## Introduction

Maternal mortality remains a serious health issue for India, and the problem for deprived parts of the population is worse. Government bodies have taken several initiatives [1], but disparities in maternal health services, provision of maternal care, and health outcomes have been noticeable across India’s socioeconomic gradient [2-4]. For example, in Kerala, the maternal mortality rate was as low as 61 per 100,000 live births in 2013, whereas in the northern states of Bihar and Jharkhand, it was 208 per 100,000 live births [5]. Interventions to minimize maternal deaths have been primarily noted to be ineffective within the rural population [6]. India’s remote parts are populous, and a lack of budget limits the success of providing adequate resources, such as health educators, infrastructure, and time to educate the targeted population [7].

Maternal mortality in rural societies of India, according to the “3 delays” model [8,9], primarily occurs due to three significant factors: (1) delay in deciding to seek care, (2) delay in obtaining timely care, and (3) delay in receiving appropriate treatment. The “3 delays” model adopts a systemic approach reflecting various obligations to avoid maternal mortality at the family, community, and health system levels. Several extrinsic factors often instigate these three delays, including insufficient support for maternal health services and the lack of awareness regarding maternal health care among pregnant women [10].

Over the years, the lack of maternal health awareness has caused several health concerns, including anemia, neural tube defects [11], tetanus infection, immunodeficiency syndrome, and even perinatal deaths [12]. Another problem faced by the tribal communities is that the people in rural India do not make use of the available health care facilities, primarily due to their belief systems. According to a previous study, approximately 21.2% of tribal women in Odisha, India, neglected their critical health conditions and sought home remedies as their primary treatment choice [13]. Certain cultures hold the extreme beliefs that their ailments, including maternal health complications, have been caused by taboos [14].

Mobile health (mHealth) technology has the potential to address the aforementioned maternal health–related concerns by augmenting maternal health awareness. Nowadays, health care is being revolutionized by mHealth. mHealth apps may have educational benefits for patients, as these technologies can offer user-friendly and easy-to-understand personalized treatment or ailment-related education [15]. Overall, mHealth for patient care can be helpful for two fundamental reasons: (1) its ability to resolve systemic obstacles (ie, language and geographical locations) and address global public health concerns [16] and (2) its promise to encourage the patient to be persistent with taking their medication [17].

Thus far, most research in this domain has been conducted in developed nations [18] and with consumers who are educated and typically familiar with mobile phones. The benefits of mHealth interventions are that they can achieve near-complete coverage of the target population. However, a large population of pregnant women in rural regions of India have no access to mobile phones [19] or any mobile technologies in general. Currently, mHealth interventions do not have sufficient evidence showing their impact on maternal health awareness, particularly in tribal and rural communities [20]. Further research may contribute to understanding the impacts of mHealth on maternal health awareness and behaviors in rural and tribal regions of India. Therefore, to address this gap, the Network for Enterprise Enhancement and Development Support (NEEDS), a nongovernmental organization (NGO) in India, and Simavi, a Dutch NGO, conceptualized and developed an easy-to-use mHealth app—Mobile for Mothers (MfM)—as a supporting tool to improve (1) maternal health awareness and (2) maternal health–related behavioral changes among tribal communities. The MfM intervention was conducted in partnership with the Rural Health Mission of the Government of Jharkhand under the European Union–funded Initiative for Transparency and Good Governance.

## Methods

### Overview

This study was part of a more extensive study conducted in collaboration with the Rural Health Mission of the Government of Jharkhand under the European Union–funded Initiative for Transparency and Good Governance. The findings from this larger study have been reported elsewhere [21-23].

### Study Design

This study was conducted with the tribal communities in rural areas of Jharkhand, India. This was a randomized quasi-controlled analysis of two groups (ie, two independent rural villages in Jharkhand, India): (1) the intervention group (village A) received maternal care via government-mandated programs through the mHealth app and (2) the control group (village B) received the same care via government-mandated programs but through traditional means (ie, verbally).

Under the government programs provided to the control group (ie, village B), community health workers, also known as accredited social health activists (ASHAs), visited each pregnant woman and discussed maternal health concerns orally, one-to-one; ensured ambulance availability if needed; and provided financial incentives for women delivering at the hospital. In the intervention group (ie, village A), ASHAs leveraged mHealth (ie, the MfM app) technology to discuss maternal health concerns and measures. All communication occurred in their native language, Hindi. Please note that participants in both groups received the same information, one-to-one; had the same number of visits; and had visits lasting a similar duration.

A total of 800 ASHAs were involved in this study, of which 400 were allocated to the intervention group. ASHAs comprise a community-based health worker group founded as a part of the National Rural Health Mission by the Indian Ministry of Health and Family Welfare. In the intervention group, ASHAs were trained in using the mHealth app (ie, MfM) for 2 days and were equipped with a Nokia phone preinstalled with the MfM app.

**Figure 1:**
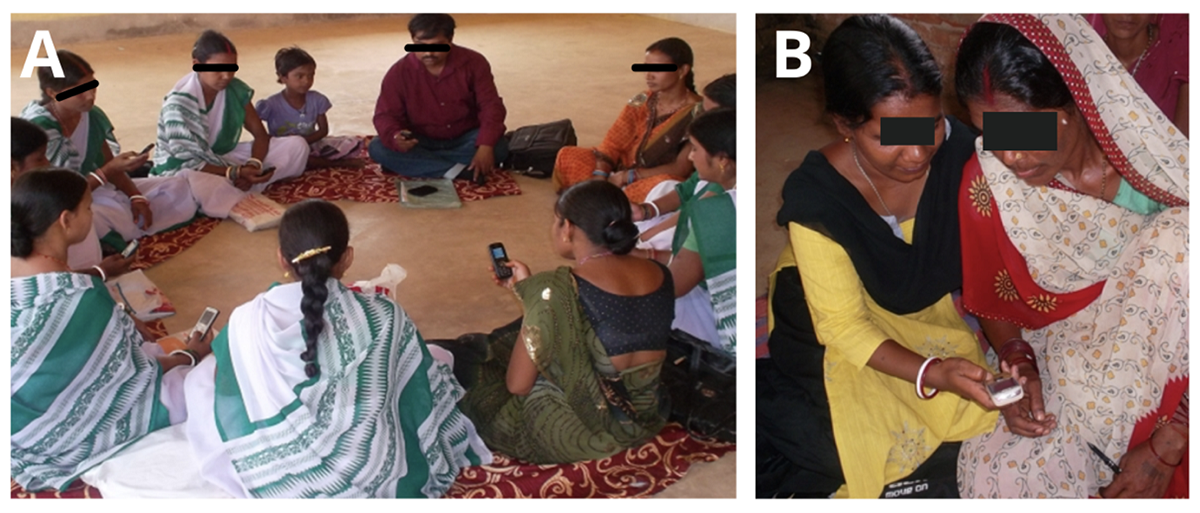
Components of the intervention. (A) Training of accredited social health activists. (B) Women using the Mobile for Mothers app as part of the intervention.

ASHAs used the MfM app to engage with pregnant women during each home visit in the intervention group. ASHA staff visited each pregnant woman four times in the prenatal phase (ie, every trimester) and twice during the postnatal phase (ie, on the third and sixth months after childbirth). During each visit, the ASHA workers used the MfM app to counsel pregnant women on antenatal care (ANC), intranatal care, and postnatal care. Figure 1 shows the training of ASHAs and their interaction with pregnant women. The control group also received the same number of AHSA visits; however, ASHA workers did not use the MfM app. Visits in both the intervention and control groups lasted approximately 45 minutes.

### Sample Size

A priori power analysis was completed to estimate the minimum sample size for the study. The a priori power analysis included 2-tailed assumptions, an estimated power of 0.80 [24], an α error probability of .01, and an effect size of 0.2 [25]. The results of the a priori power analysis supported the inclusion of at least 1172 participants in the study.

### The Mobile for Mothers App

**Figure 2:**
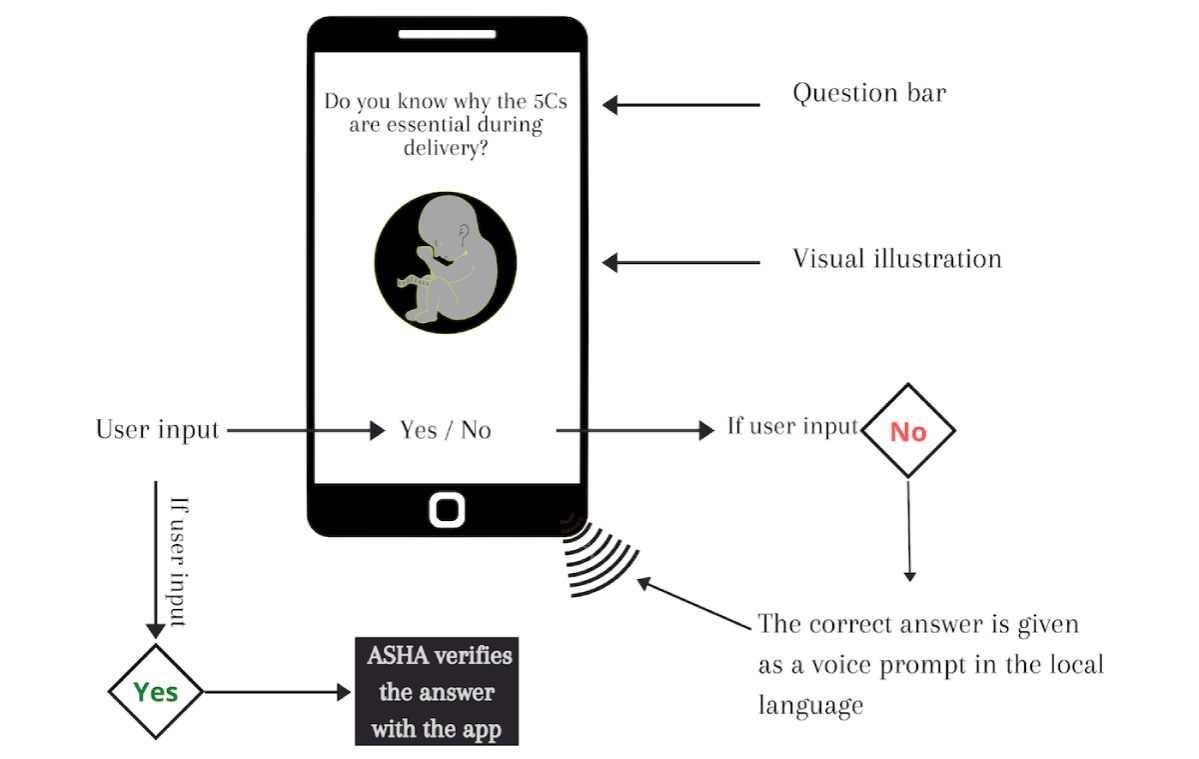
The Mobile for Mothers mobile health app. The original app was in the Hindi language. 5C: five cleans (clean hands, clean place, clean cloths, clean cord cut with a clean blade, and clean cord clamped with a clean thread); ASHA: accredited social health activist.

MfM is a case management solution in rural India for ASHAs. The program helps handle the registration, service, and monitoring of all clients and events related to the ASHAs. MfM collects data from each home visit and transfers the data for service optimization, wellness surveillance, and workflow measures to the NEEDS management committee. MfM is designed for low-literacy users so they can operate the app on affordable Java-enabled phones or Android-based smartphones that run free and open-source apps. It contains registration forms, checklists, tracking of danger signs, and an interactive voice-recording system for instructional prompts. In addition, the app provides maternal health information through texts, photographs, and voice prompts in their native language of Hindi to pregnant women and mothers, as illustrated in Figure 2.

### Data Collection

In January 2014, researchers conducted a complete house list and identified eligible respondents: all pregnant women between 18 and 45 years of age. Soon after, a team of trained project members, who were local to the region, administered a paper-based survey in the control and intervention villages to collect baseline data. During the survey, team members read the questions in Hindi and marked the responses for all the participants, including illiterate and literate pregnant women. The survey used in this study was based on The National Family Health Survey [26] and measured the pregnant women’s maternal health and hygiene awareness.

The mHealth intervention commenced soon after the baseline survey was completed in February 2014. The end-line data were collected between November 2015 and January 2016 by the same project members and in the same manner.

### Outcome Measures

Maternal health awareness and related behavioral changes were captured in this study. Awareness was calculated as a binary variable where women were deemed to be aware of maternal health information if they responded “yes,” followed by the correct explanation, to the following awareness questions:

1. Do you know why the five cleans (5Cs: clean hands, clean place, clean cloths, clean cord cut with a clean blade, and clean cord clamped with a clean thread) are essential during delivery?
2. Pregnant women should receive two tetanus vaccine injections. There should be a difference of 1 month between the first and second injections. Does this protect you and your baby from tetanus?
3. Do you know that if you have problems like painful or burning urination and itchy genitals during pregnancy they are indicative of a reproductive tract infection?
4. Do you know why you need to test for HIV/AIDS during or before pregnancy?
5. Have you consumed all of your iron tablets?

The ASHAs determined the validity of their responses. The MfM app also prompted the correct answers in Hindi.

### Data Analysis

Responses from the baseline and end-line paper-based surveys were manually entered into a Microsoft Excel sheet by two research assistants (NEEDS staff). After verifying the data entry, all data were imported into SPSS software (version 27; IBM Corp) for further analysis.

First, we calculated descriptive statistics related to demographics. The percentage changes for each variable between baseline and end line in each group were also calculated. The baseline preintervention groups were compared to the end-line postintervention groups using Pearson chi-square analyses, showing the significance of pre- to postintervention differences (ie, percentage changes) within each group. In addition, Mantel-Haenszel tests for conditional independence were conducted to determine if the pre- to postintervention differences in the intervention group were significantly different (at a 99% CI) from the pre- to postintervention differences in the control group.

### Ethics Approval

This study obtained ethical approval from the Institutional Review Board of the Centre for Media Studies, New Delhi, India (approval No. IRB00006230). Given the low level of literacy among the sample population, verbal consent was acquired rather than written documentation. Researchers read the consent form in Hindi. Before the study began, all participants were briefed on the in-depth intent of the study. No participant identifiers were obtained during the study.

## Results

The survey consisted of 1480 respondents, with 740 women per group. In total, 73.2% (542/740) and 70.7% (523/740) of the respondents in the intervention and control groups, respectively, belonged to other backward castes. In both groups, about half of the respondents were illiterate. Most women in both study groups were housewives. Table 1 shows the participant demographics.

**Table 1:** Demographic characteristics of the study participants (N=1480).

Demographic characteristic			Control village (n=740), n (%)		Intervention village, (n=740), n (%)
**Caste**					
	Scheduled caste	131 (17.7)		100 (13.5)	
	Scheduled tribe	22 (3.0)		29 (3.9)	
	Other backward castes	523 (70.7)		542 (73.2)	
	Other than scheduled cast, scheduled tribe, or other backward castes	63 (8.5)		69 (9.3)	
**Educational level**					
	Illiterate (never been to school)	384 (51.9)		385 (52.0)	
	Primary (1-5 years of schooling)	124 (16.8)		141 (19.1)	
	Secondary (6-10 years of schooling)	199 (26.9)		187 (25.3)	
	Higher (≥11 years of schooling)	33 (4.5)		29 (3.9)	
**Occupational status**					
	Housewife	684 (92.4)		645 (87.2)	
**Age during intervention (years)**					
	18-19	135 (18.2)		145 (19.6)	
	20-24	383 (51.8)		369 (49.9)	
	25-29	160 (21.6)		158 (21.4)	
	30-34	47 (6.4)		47 (6.4)	
	35-45	15 (2.0)		21 (2.8)	
**Age at marriage (years)**					
	<18	470 (63.5)		501 (67.7)	

As shown in Table 2, except for awareness regarding tetanus vaccine injections, we noted significant improvement, from baseline to end line, in maternal health awareness in both the intervention and control groups. However, the magnitude of improvement was significantly higher in the intervention group.

Awareness regarding the 5Cs in the intervention group increased (*P*<.001) from among 143 out of 740 participants at baseline to 555 out of 740 participants at end line. In contrast, in the control group, awareness increased (*P*<.001) from among 108 out of 740 participants at baseline to 555 out of 740 participants at end line. However, the increase in awareness was significantly greater in the intervention group than in the control group (*P*<.001).

Awareness about tetanus vaccine injections and the fact that pregnant women should receive two shots of tetanus vaccine were significantly increased (*P*<.001) from among 73 out of 740 participants to 372 out of 555 participants in the intervention group. However, for the control group, awareness increased (*P*<.001) from among 39 out of 108 participants to 220 out of 492 participants. The magnitude of improvement for tetanus vaccine awareness was also significantly higher in the intervention group than in the control group (*P*<.001).

In the intervention group, awareness regarding the fact that problems like painful or burning urination and itchy genitals during pregnancy are indicative of a reproductive tract infection (ie, vaginal yeast infection) increased (*P*<.001) from among 15 out of 740 participants to 608 out of 740 participants. However, in the control group, awareness increased (*P*=.10) from among 7 out of 740 participants to 132 out of 740 participants. The magnitude of improvement regarding the awareness of reproductive tract infection was also significantly higher in the intervention group than in the control group (*P*<.001).

Similarly, knowledge about HIV testing increased (*P*<.001) from among 39 out of 740 participants to 572 out of 740 participants in the intervention group. In contrast, in the control group, knowledge increased from among 28 out of 740 participants to 131 out of 740 participants. The change in HIV-related awareness was significantly greater among the intervention group than among the control group (*P*<.001).

Since awareness can be a precursor to behavioral change, we noted that the number of pregnant women in the intervention group who consumed the prescribed dosage of iron tablets increased (*P*<.001) from 193 out of 288 participants at baseline to 612 out of 663 participants at end line. However, the number of pregnant women in the control group who consumed the prescribed dosage of iron tablets increased (*P*<.001) from 129 out of 212 participants at baseline to 223 out of 297 participants at end line. The magnitude of behavioral improvement for iron consumption was significantly higher in the intervention group than in the control group (*P*<.001).

**Table 2:** Comparison of responses to awareness questions by the intervention and control group participants.

Question and group			Baseline, n (%)^a^		End line, n (%)^b^		Change from baseline to end line, %		Comparison of baseline and end line					Comparison of change between groups		
									*χ* ^2^_1_		*P* value		*χ* ^2^_1_			*P* value
**Do you know why the five cleans are essential during delivery?^c^**																
	Control	108 (14.6)		492 (66.5)		51.9		413.3		<.001		—^d^			—	
	Intervention	143 (19.3)		555 (75.0)		55.7		460.3		<.001		870.5			<.001	
**Pregnant women should receive two tetanus vaccine injections. There should be a difference of 1 month between the first and second injections. Does this protect you and your baby from tetanus?^e^**																
	Control	39 (36.1)		220 (44.7)		8.6		2.7		.10		—			—	
	Intervention	73 (9.9)		372 (67.0)		57.1		459.4		<.001		380.3			<.001	
**Do you know that if you have problems like painful or burning urination and itchy genitals during pregnancy they are indicative of a reproductive tract infection?^c^**																
	Control	7 (0.9)		132 (17.8)		16.9		124.1		<.001		—			—	
	Intervention	15 (2.0)		608 (82.2)		80.2		974.8		<.001		1055.6			<.001	
**Do you know why you need to test for HIV/AIDS during or before pregnancy?^c^**																
	Control	28 (3.8)		131 (17.7)		13.9		74.8		<.001		—			—	
	Intervention	39 (5.3)		572 (77.3)		72.0		791.9		<.001		804.8			<.001	
**Have you consumed all of your iron tablets?^f^**																
	Control	129 (60.8)		223 (75.1)		14.3		11.8		.001		—			—	
	Intervention	193 (67.0)		612 (92.3)		25.3		98.8		<.001		87.7			<.001	

^a^The number of respondents in this column represents the number at baseline who responded “yes” to the questions.

^b^The number of respondents in this column represents the number at end line who responded “yes” to the questions.

^c^Control and intervention group participants at baseline and end line: n=740.

^d^Statistics for group comparisons are reported only in the intervention group row.

^e^Control group participants at baseline: n=108; control group participants at end line: n=492; intervention group participants at baseline: n=740; intervention group participants at end line: n=555.

^f^Control group participants at baseline: n=212; control group participants at end line: n=297; intervention group participants at baseline: n=288; intervention group participants at end line: n=663.

## Discussion

### Principal Findings

When most people are demonstrating techno-skepticism (ie, a skeptical attitude toward technology), in our study, pregnant women residing in tribal societies exhibited characteristics of the “learned skeptic.” However, in augmenting their awareness, these tribes learned maternal health lessons efficiently from the MfM app. In addition, the results of our study indicate the potential of MfM specifically, or mHealth in general, as an educational tool for tribal communities.

Existing literature has acknowledged several mobile apps that can assist with maternal education and support socially disadvantaged pregnant women [27]. Despite having minimal prior experience with mobile devices and no health literacy, our study demonstrates how tribal communities learned information about maternal health and hygiene when delivered through the mHealth app in a user-centered manner (ie, using their regional language and visuals). The MfM intervention also improved the pregnant women’s health behavior, in the form of being persistent with iron supplementation. Persistence is measured by how an individual’s conduct meets the desired health care objectives jointly identified with the health care professional [28]. Overall, our study indicates that the mode of information transference can determine its acceptance by users and their likelihood of gaining new knowledge. According to the cognitive theory of multimedia learning, people learn more effectively from words and images than from words alone [29]. This theory can partially explain why tribal communities learned new information through MfM significantly more than through traditional government interventions in which information transference was verbal. Using both auditory and visual channels in MfM (ie, dual-coding theory) perhaps helped pregnant women acquire new knowledge. However, these assumptions require further confirmation.

### Maternal Health Awareness

Lack of maternal health awareness is a significant concern in rural and tribal India, causing several health concerns, such as neural tube defects [10] and restricted fetal growth [30]. Poor awareness regarding hygiene (ie, the 5Cs) and tetanus, a life-threatening bacterial disease, have also been responsible for genital tract infection (ie, vaginal yeast infection), puerperal sepsis, and even morbidity among rural and tribal communities [31-33]. Our study demonstrated that mHealth, like MfM, could augment health awareness regarding the 5Cs and tetanus. In our research, more pregnant women in the intervention group acknowledged that the 5Cs can prevent infection in newborn babies and mothers.

Poor awareness has also resulted in tetanus infections and immunodeficiency syndromes, causing harm to pregnant women and newborn children [34,35]. In pregnant women, reproductive tract infection risk predominantly occurs due to a lack of awareness and can lead to postabortal sepsis, puerperal sepsis, and even perinatal death [11]. Deaths of pregnant women due to HIV in rural communities are often induced by poor pregnancy management, primarily due to the lack of knowledge about HIV infection [36].

Our findings were consistent with other studies concerning mHealth and maternal health, where increased awareness was reported. A 2014 study in India’s rural region evaluated the impact of mHealth on raising awareness regarding maternal health. The study found that more individuals learned about danger signs during pregnancy, such as infections, after receiving text messages from the mHealth app [37]. Another study in Nigeria investigated the impact of mHealth on maternal knowledge. The study reported that pregnant women without mHealth access had significantly lower knowledge of maternal danger signs than those with access to mHealth apps [38].

Unlike our study, where mHealth increased awareness regarding HIV and tetanus among tribal pregnant women, a randomized controlled trial implementing mHealth to augment maternal health awareness did not find any significant increase in awareness regarding tetanus and HIV [39].

### Iron Tablet Consumption

Exposure to an interactive voice recording system for instructional prompts, like MfM, could improve a pregnant woman’s health behaviors. For example, in our study, more pregnant women in the intervention group than in the control group consumed the prescribed dosage of iron tablets (25%; *P*<.001). Other studies also reported similar findings. In 2014, a study in Kenya reported that pregnant women (91.6%) receiving an mHealth intervention consumed the necessary dosage of supplements [39]. In 2017, a study in India registered a significant increase in the consumption of iron supplements among pregnant women (81%) receiving mHealth support [40]. Similarly, another study conducted in Indonesia reported a 2.6% increase in the consumption of iron tablets among pregnant women receiving an mHealth intervention [41].

### Limitations

This study only focused on two villages; though the sample size was sufficient, our findings may not be generalizable across all tribal and rural communities. Due to limited access to mobile phones in rural India, ASHA workers were responsible for carrying the mobile device with them during each intervention. Pregnant women, being passive users, only used the mobile app in the presence of the ASHAs. Further research is needed to capture the direct impact of mHealth on maternal health awareness and behavior in tribal communities when pregnant women are active users without assistance from trained personnel such as ASHAs.

### Implications, Future Directions, and Conclusions

Although improvements in maternal health care awareness were observed in both control and intervention groups, our study provides evidence that mHealth interventions can improve critical maternal health awareness and related behavior among tribal pregnant women at a significantly higher magnitude. Our analysis also exhibited the potential of MfM to minimize the cognitive biases of tribal communities. Tribal communities often show anchoring bias. In other words, their dependency on prior knowledge (ie, “traditional health care system”) [42] is significant. Their health care practices and beliefs are primarily determined and constructed by their faith in traditional knowledge, which involves three ways of addressing health complications—natural medicine, psychosomatic treatments, and religious rituals—that typically reject the intervention of scientific methods [42]. However, participants in the intervention group behaved otherwise. Despite firmly believing in the “traditional health care system,” tribal communities in the intervention group were noted to break away from their traditional beliefs and adhere to scientific or modern maternal health care practices.

The findings of our study exhibit the effectiveness of mHealth and encourage the use of mHealth as a supporting tool for health workers serving in rural regions. In addition to augmenting maternal health awareness and behavior, mHealth interventions may lower health care costs in several ways, including transportation cost reduction for patients and digitizing data collection (ie, registration of pregnant women, tracking their ANC services, and others). Moreover, in urban societies where pregnant women have access to mobile phones, the internet, and sufficient ability to use smartphones independently, mHealth apps can serve as an alternative source of maternal health information. In 2013, a study reported that about 40% of its 35 participants had used at least one mHealth app for maternal information [43]. Another study in 2016 surveyed 410 women and found that 92% of users found mHealth to be useful for pregnancy-related information [44]. Therefore, future studies should consider evaluating the economic feasibility of mHealth in maternal health awareness [45].

## Appendix

Multimedia Appendix 1 CONSORT checklist.

## Abbreviations

5Cs: five cleans (clean hands, clean place, clean cloths, clean cord cut with a clean blade, and clean cord clamped with a clean thread)
ANC: antenatal care
ASHA: accredited social health activist
MfM: Mobile for Mothers
mHealth: mobile health
NEEDS: Network for Enterprise Enhancement and Development Support
NGO: nongovernmental organization

## Data Availability

Deidentified end-line data will be made available only for research purposes upon request.
